# Effects of a daylight intervention in the morning on circadian rhythms and sleep in geriatric patients: a randomized crossover trial

**DOI:** 10.1007/s41999-024-01100-z

**Published:** 2024-12-03

**Authors:** Anna Schubert, Thea Laurentius, Svenja Lange, Jens Bertram, Leo Cornelius Bollheimer, Marcel Schweiker, Rania Christoforou

**Affiliations:** 1 Healthy Living Spaces Lab, Institute for Occupational, Social and Environmental Medicine, Medical Faculty, RWTH Aachen University, Pauwelsstraße 30, 52074 Aachen, Germany; 2 Department of Geriatric Medicine, RWTH Aachen University Hospital, 52074 Aachen, Germany; 3 Institute for Occupational, Social and Environmental Medicine, Medical Faculty, RWTH Aachen University, 52074 Aachen, Germany

**Keywords:** Sleep, Circadian rhythm, Melatonin, Cortisol, Subjective sleep quality, Actigraphy

## Abstract

**Purpose:**

During hospitalization, circadian rhythms and sleep are often disrupted, which has negative effects on health outcomes. Therefore, we aimed to investigate whether a daylight intervention in the morning could improve the circadian rhythms of cortisol and melatonin and enhance objective and subjective sleep quality in geriatric patients.

**Methods:**

The present study is a randomized, two-period crossover trial conducted in a geriatric ward in 15 non-demented geriatric trauma patients with a mean age of 83.1 ± 5.4 years. All patients underwent a daylight intervention period, during which they were exposed to a daylight lamp from 8:00 to 13:00 h, and a control period of 6 days each. Cortisol and melatonin levels were measured on day 5 of each period. Objective and subjective sleep quality were assessed using actigraphy and questionnaires, respectively. Within-participant differences between periods were investigated for all parameters.

**Results:**

A trend towards improvement in cortisol and melatonin rhythmicity was found. An increase in mean melatonin levels from 0.3 ± 0.1 to 0.9 ± 0.8 ng/L was observed during the intervention period (*p* = .063). There was also a trend towards increased sleep efficiency, whereas subjective sleep quality tended to decrease. None of the results were significant.

**Conclusion:**

A daylight intervention in the morning led to a positive trend in cortisol and melatonin rhythmicity, whereas no improvement in subjective sleep quality was found.

**Trial registration:**

DRKS00028626 at German Clinical Trials Register, 13.06.2022.

**Supplementary Information:**

The online version contains supplementary material available at 10.1007/s41999-024-01100-z.

## Key summary points

**Aim:**

To explore whether a daylight intervention could improve the circadian rhythms of cortisol and melatonin in geriatric patients, alongside their sleep quality.

**Findings:**

The results indicated a tendency towards an enhancement of the endocrinological parameters' circadian rhythms. However, there was no improvement in subjective sleep quality following the intervention.

**Message:**

A daylight intervention could be of value in enhancing the circadian rhythms of geriatric patients.

**Supplementary Information:**

The online version contains supplementary material available at 10.1007/s41999-024-01100-z.

## Introduction

Sleep during hospitalization is neither subjectively nor objectively adequate [1]. More than 40% of patients report poor subjective sleep quality (S-SQ), which can be defined as “an individual's self-satisfaction with all aspects of the sleep experience” [1, 2]. Objectively viewed, hospitalization reduces total sleep time (TST) on average from 7.1 to 5.3 h [3]. Sleep reduction can be highly problematic, as sleep deprivation and disruption are associated with poorer health outcomes such as an increased risk for delirium, and even an increased mortality risk in geriatric patients [4–6]. It is therefore important to increase sufficient sleep during hospitalization.

Sleep disorders can be caused by disturbed circadian rhythms [7]. Circadian rhythms are internal rhythms with a period length of about 24 h that are influenced by endogenous and external factors. These factors strongly control sleep–wake patterns and other physiological functions like the release of hormones [1, 4]. Both the hormones cortisol and melatonin are linked to the sleep–wake cycle and are themselves subject to circadian rhythms. Cortisol peaks shortly after waking in the morning and reaches its minimum in the late evening [8]. Melatonin levels are typically low during the day and peak in the middle of the sleep period [8]. The main Zeitgeber for circadian rhythms is light [9]. Both sufficient light exposure during the day and no nocturnal exposure are important for entraining and maintaining circadian rhythms [9]. Neither is given in hospital surroundings [1]. Even hospitalized patients without a history of sleep disorders can suffer from circadian disruption during hospitalization and afterwards [9].

The oldest old (≥ 85 years) are the fastest growing population, being hospitalized more often than younger patients [10, 11]. It is important to maintain or restore circadian rhythms and prevent sleep problems associated with hospitalization in older people as they are especially vulnerable to sleep deprivation and disruption. These sleep related deficits have been reported to be independent risks of mortality for geriatric patients [5, 6]. At the same time, the majority of sleep medications currently available are potentially inadequate medications for older people [12].

Previous studies have shown that light therapy can be effective in treating sleep disorders, although most effects are mild to moderate and results across studies are inconclusive [9, 13]. Therefore, a light intervention could improve geriatric patients' circadian rhythms and sleep without the need for additional sleep medication. A morning light intervention has been demonstrated to be particularly beneficial for geriatric patients, as evidenced by the findings of Wakamura et al., Kobayashi et al., and Fukuda et al. [14–16].

In the present study, we aimed to investigate whether a daylight lamp intervention in the morning could improve the circadian rhythms of cortisol and melatonin and enhance S-SQ and objective sleep quality (O-SQ) in geriatric patients. Additionally, it was hypothesized that geriatric patients will sleep better objectively and subjectively over the course of their hospital stay, as their condition improves. A secondary hypothesis was that S-SQ and O-SQ will be correlated [17, 18].

## Methods

### Study design

**Fig. 1:**
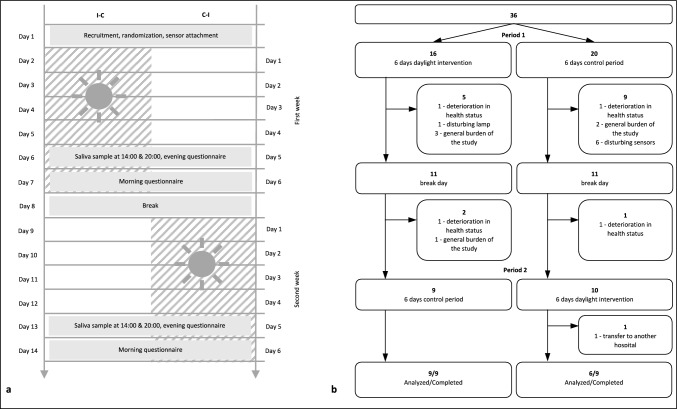
**a** Illustration of the crossover design showing the study schedule for both sequence groups. One group received the intervention in the first week (days 2–7), while the other received it in the second week (days 9–14). Saliva samples were taken on days 6 and 13. There was a study break on day 8. *I-C* intervention-control group, *C-I* control-intervention group*.* **b** Participant flow. 36 patients were included and randomized, 22 completed the first week, 18 completed the study, and 15 were included in the analysis

The study was designed as a crossover study, since a randomized-controlled trial would have required a high, unfeasible number of cases, considering the great heterogeneity of geriatric patients. There were two periods, lasting 6 days each, with a washout period of 1 day between them (Fig. 1). Subjects were alternately assigned to the sequence groups by a study investigator. As the study progressed, participants were assigned in such a way that the sequence groups remained the same size despite the dropouts. Neither the participants nor the investigators could be blinded. The laboratory tests of cortisol and melatonin levels were performed in a blinded manner.

The study period covered only the winter months from November 2022 to early April 2023, to have comparable environmental conditions, such as similar natural day length and weather.

The study protocol was approved by the local ethics committee of the medical department at RWTH Aachen University (No. EK166/22). All participants gave their written consent.

### Subjects

Due to a lack of comparable studies, a pragmatic approach was adopted to establish an appropriate sample size. After considering various clinic-related factors, including patient throughput and patient characteristics, we determined that conducting the study with 15–20 patients within a 6-month time frame was feasible.

The 36 subjects enrolled, of whom 15 were analyzed, were geriatric traumatological patients recruited from the geriatric ward of the RWTH Aachen University Hospital. They had to be at least 70 years old, and their planned hospital stay had to be 14 days or longer.

Exclusion criteria were an active infection at the start of the study or a cognitive impairment, measured with the Mini-Mental Status (MMS) examination [19]. The cut-off was an MMS < 25.

### Interventions

On the first day, subjects were recruited by a study investigator and assigned to the sequence groups, and sensors were attached. No intervention took place. On day two, either the control period (CP) or intervention period (IP) started, lasting from days 2 to 7. Day 8 was a break day used as washout period to prevent effects from the first period being carried over into the second. The subjects kept wearing the sensors, but no data were assessed. On day 9, the CP or IP started depending on which period was completed in the first study week, as shown in Fig. 1a.

During the CP, the circumstances in the patients’ surroundings were not changed. In the IP, a daylight lamp was placed on the bedside table. It was turned on at 8:00 h ± 10 min and turned off at 13:00 h ± 10 min by study staff. When assessed with a spectroradiometer by Apacer (Ai101 spectral irradiance meter, Apacer Technology Inc., New Taipei City, Taiwan, R.O.C, https://iiot.apacer.com/tw/solutions/ai101/) at less than 5 cm distance, the daylight lamp had an illuminance of 14,762 lx, a dominant wavelength of 486 nm, and a color temperature of 4063 K.

### Measurements

Primary outcomes were cortisol and melatonin levels measured on day 5 of each period and S-SQ. Secondary outcomes were O-SQ and light exposure.

#### Measurement of cortisol and melatonin

On day 5 of each condition, saliva samples for measuring cortisol and melatonin levels were taken at 14:00 h and 20:00 h. Times were chosen based on the circadian rhythms of the hormones with the aim to identify potential hormone peaks resulting from the light intervention in the morning. To obtain the highest and, respectively, lowest levels, it was planned to take the nighttime samples at 23:00 h. This led to sleep disruption, which was avoided by adjusting the saliva sampling time from 23:00 to 20:00 h. Nighttime samples were only included in analyzes when collected at 20:00 h. The lighting during the saliva collection was only changed by switching on a lamp in the entrance area of the room if it was completely dark.

Before sample collection, a 1-h fasting period was ensured and subjects were asked to rinse their mouth with saline for at least 10 s to avoid contamination with food, beverage, or drug residues. Saliva was collected in SALIVETTE^®^ tubes (SALIVETTE^®^, Sarstedt, Nümbrecht, Germany, https://www.sarstedt.com/produkte/diagnostik/salivasputum/produkt/51.1534/). Subjects kept the sample carrier in their mouths for 2 min without chewing, according to SALIVETTE^®^ instructions. Procedures were monitored throughout, and when considered appropriate, they were modified accordingly. Following the observation that 2 min were too short for collecting the needed saliva amount for analysis, the subjects were asked to keep the sample carrier in their mouths for 10–15 min. Samples were stored in SALIVETTE^®^ tubes at − 21 °C until analysis. The analysis was based on the liquid chromatography–tandem mass spectrometry method of Jensen et al. [20]. In brief, 250 µL of saliva or less were diluted in 0.5 mM ammonia acetate pH 7.5 and with isotope labelled deuterated internal standards of d4-cortisol (Merck, Darmstadt, Germany) and d4-melatonin (TRC, Toronto, Canada). After adding 1 mL ethyl acetate, the samples were shaken for 45 min for liquid–liquid extraction. The supernatant was concentrated and diluted again to a final volume of 100 µL and injected into the LC/MSMS-system (QTRAP 5500, ABSciex, Darmstadt, Germany). We collected saliva using SALIVETTE^®^ tubes rather than from drool, which has no impact on the results [21]. The detection limits were 0.1 µg/L for cortisol and 0.1 ng/L for melatonin. For comparisons with other studies, saliva melatonin levels are assumed to correspond to 30% of the plasma levels [22].

#### Evaluation of S-SQ

S-SQ was assessed daily at 8:00 h ± 10 min by study staff. Subjects were asked “How well did you sleep last night?” [Rated on a scale of 0 (very bad) to 10 (very good)]. The scale was explained to the patients daily.

On days 5 and 6 of both weeks, additional questionnaires were completed assessing subjective diurnal sleep duration, use of sleep medication, restfulness of sleep and mood after waking up (supplementary information (SI) “Supplementary file 1”).

#### Actigraphy (O-SQ)

Sleep-related behavior was recorded objectively by actigraphy. An accelerometer (Actigraph wGT3X-BT, Actigraph Corp., Pensacola, FL, https://theactigraph.com/actigraph-wgt3x-bt) was attached to the subjects using a belt around either their hip or thigh, depending on their physical constitution. It was worn directly on the skin throughout the whole study period except during showering. Accelerations were recorded at a 30 Hz sampling rate.

#### Measurement of light exposure

The actual light exposure was assessed with the MSR145 data logger (MSR145 data logger, MSR Electronics GmbH, Seuzach, Switzerland, https://www.msr.ch/de/produkt/datenlogger-temperatur-feuchte-druck-beschleunigung-msr145/). It was worn on the upper arm of participants, over their clothing, for the whole study period except during showering.

#### Participant characteristics

Demographic data, clinical data, and geriatric assessment were acquired from patient records.

### Data processing and analysis

Data from the MSR145 data logger were processed using MSR software (version 6.03.02, MSR Electronics GmbH, Seuzach, Switzerland).

Actigraphy data were processed using Actilife software (version 6.13.4, Actigraph Corp., Pensacola, FL) and considerations by Migueles et al. were used as guidance for analysis [23]. Actilife’s low-frequency extension filter was applied to all data and an epoch length of 60 s was used. Before wear time validation and sleep analysis were performed, a clinical wear time period was established during which most participants wore the Actigraph, as no clinical wear time of 100% was reached for all subjects. The clinical wear time period was defined as day 1, 8:00 h to day 5, 13:00 h (4 days 5 h) for each week. Actigraphy data were truncated to this period. Wear time was defined as valid if the Actigraph was worn for at least 80% per day in the clinical wear time period. Wear time validation was done by overlapping wear times calculated using the Choi algorithm with wear times detected by Actigraph’s wear sensor, an integrated capacitive proximity sensor [24]. Using the wear sensor data alongside the data obtained from the Choi algorithm allows a more precise estimation of wear time, especially for individuals confined to bed. A wear period was defined if it was identified as such by the Choi algorithm, the wear sensor, or both.

Sleep periods were detected through Actilife’s algorithm. The Cole–Kripke algorithm was used for calculating sleep efficiency (SE, proportion of total time in bed actually spent sleeping), sleep onset latency (SOL, time from turning off the light to falling asleep), TST, wakefulness after sleep onset (WASO), and sleep fragmentation [25]. Additionally, sleep periods were divided into diurnal and nocturnal sleep periods and diurnal or nocturnal total sleep time (TSTD, TSTN), respectively, was calculated. Nocturnal sleep periods were defined as sleep periods starting between 20:00 and 4:30 h, and diurnal sleep periods as sleep periods starting between 4:31 and 19:59 h. For further analysis, average values of all sleep periods of IP or CP, respectively, were used for all sleep parameters.

Statistical analysis was done with IBM^®^ SPSS^®^ Statistics (IBM Corp. Released 2022. IBM SPSS Statistics for Windows, Version 29.0. Armonk, NY: IBM Corp). A *p* value of < 0.05 was considered significant.

Differences between sequence groups in basic demographic data were calculated using an independent two-sample *t* test for parametric data, a Mann–Whitney *U* test for non-parametric data, and a Chi-square-test for categorical variables.

Any outliers were treated individually. For endocrinological parameters, outliers were only removed from further analyses if they were outside the normal range [22, 26]. For O-SQ, outliers were removed if they could be caused by limited mobility, which leads to an overestimation of the sleep time recorded using actigraphy [27].

Before conducting the main analyses, data were checked for missing completely at random (MCAR) [28]. If data were MCAR, data were imputed with expectation maximization techniques if ≤ 6.7% (1 out of 15) were missing. Otherwise, data were deleted pairwise and excluded from the analysis.

Preliminary tests were conducted to test for period effects [29]. There was no need to adjust main analysis for period effects. According to Lim et al. and Senn et al., a washout phase of 1 day was interposed rather than testing for carry-over effects [29, 30].

For the intervention effect, paired *t* tests for parametric data and Wilcoxon’s tests for non-parametric data were performed.

Graphical trend analyses were performed to explore variations in S-SQ between the days.

Correlation analyses were conducted between S-SQ and actigraphy using Spearman’s rank correlation coefficient.

## Results

A total of 36 patients were enrolled in the study; 18 dropped out. Participant flow is shown in Fig. 1b. Reasons for drop out included withdrawing consent due to the general burden of participating in a clinical trial (*n* = 6), considering the lamp (*n* = 1) or sensors disturbing (*n* = 6), deterioration in health status (*n* = 4), and transfer to another hospital (*n* = 1). There were no adverse events.

Six subjects were analyzed in the C-I group, nine subjects in the I-C group, with one participant per group being discharged prior to completing the second week. Three subjects in the C-I group were excluded from all analyses as they began taking melatonin as a sleeping aid during the study period.

Two subjects in the C-I group were using mirtazapine as a sleeping aid at study entry and continued using it for the entire period. In the I-C group, no subjects used sleeping aids during the study. All subjects used non-opioid pain medication; 11 subjects were treated with opioids.

Baseline demographic and clinical characteristics and geriatric assessment are shown in Table 1. There were no significant differences between the two sequence groups.

**Table 1:** Baseline demographic and clinical characteristics and geriatric assessment by sequence and by total

	I-C (*n* = 9)	C-I (*n* = 6)	Mean difference (95% CI)	*p* value	Total (*n* = 15)
*Sex*^a^				0.26	
Male	2 (22)	3 (50)			5 (33)
Female	7 (78)	3 (50)			10 (66)
Age (years)^b^	84.1 ± 5.5	81.7 ± 5.2	– 2.4 (– 8.6, 3.7)	0.41	83.1 ± 5.4
Weight (kg)^b^	61.2 ± 9.1	61.4 ± 9.2	0.1 (– 10.3, 10.5)	0.98	61.3 ± 8.8
Height (cm)^b^	168 ± 7	164 ± 11	– 4.0 (– 15.2, 7.2)	0.44	167 ± 0.0
BMI (kg/m^2^)^b,1^	21.9 ± 3.2	22.7 ± 2.2	0.8 (– 2.8, 4.4)	0.67	22.1 ± 2.8
Days since hospitalization^c^	2 ± 1.8	1 ± 1.4	– 0.4 (– 2.3, 1.4)	0.78	2 ± 1.6
*Main diagnosis (ICD-10-GM Version 2024)*^a^				0.31	
R29.6	1 (11)	2 (22)			3 (20)
S22.-	1 (11)	0 (0)			1 (7)
S32.-	3 (33)	0 (0)			3 (20)
S42.-	0 (0)	1 (11)			1 (7)
S72.-	3 (33)	3 (33)			6 (40)
T84.-	1 (11)	0 (0)			1 (7)
*Treatment of the primary disease*^a^				0.40	
Conservative	5 (56)	2 (20)			7 (47)
Operation	4 (44)	4 (80)			8 (53)
Days since operation^c^	5 ± 1.7	7 ± 1.7	1.8 (– 1.2, 4.7)	0.20	6 ± 1.8
Analgesic treatment with opioids^a^	7 (78)	4 (66)		0.63	11 (73)
*Geriatric assessment*					
De Morton Mobility Index^c^	33.3 ± 7.3	40.0 ± 12.5	6.3 (– 7.0, 19.6)	0.33	35.9 ± 9.8
Geriatric depression score^c^	3.1 ± 2.3	1.4 ± 0.9	– 1.7 (– 3.6, 0.2)	0.30	2.5 ± 2.1
Mini-mental status^c, 2^	26.0 ± 1.3	28.7 ± 4.0	2.7 (– 6.9, 12.2)	0.80	26.7 ± 2.5
Hand grip of dominant hand (kg)^b,3^	15.4 ± 5.5	19.4 ± 9.0	4.0 (– 4.4, 12.3)	0.32	16.9 ± 6.9
Clinical frailty score^c^	2.3 ± 1.3	1.8 ± 0.8	– 0.5 (– 1.7, 0.7)	0.53	2.1 ± 1.1

Values are presented as mean ± SD or total number (%). *I-C* intervention-control group, *C-I* control-intervention group

^a^Chi-square test

^b^Independent two-sample *t* test

^c^Mann–Whitney *U* test

^1^*n* = 12 (I-C: 8, C-I: 4)

^2^*n* = 11 (I-C: 8, C-I: 3)

^3^*n* = 14 (I-C: 9, C-I: 5)

### Wear time validation

Clinical wear time of the Actigraph was met by 12 out of 15 subjects (I-C: 6, C-I: 6), and valid wear time by 10 subjects (I-C: 4, C-I: 6).

### Variables by period

There were no significant period effects (SI, “tables”, Table SI-1).

**Fig. 2:**
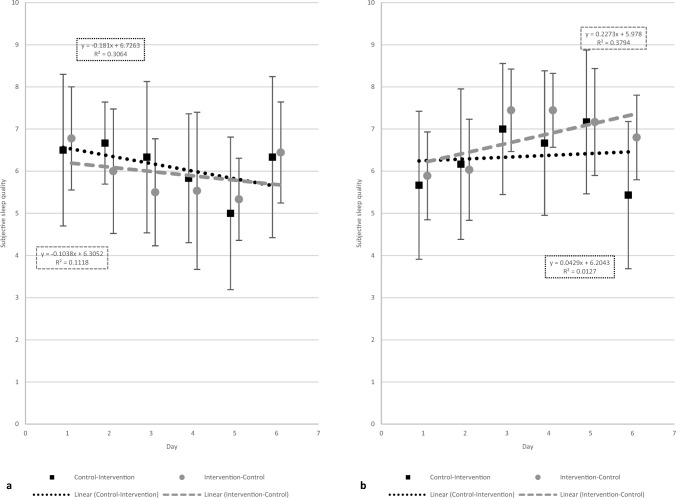
Mean S-SQ in the first week (**a**) and in the second week (**b**). **a** S-SQ tends to decrease in the first week. The intervention-control group receiving intervention here demonstrated a comparatively smaller decline in S-SQ in contrast to the control-intervention group. **b** In the second week, the S-SQ in the control-intervention group remained almost constant over the course of the intervention. The S-SQ of the intervention-control group, which received no intervention during that week, showed a positive trend

Figure 2 shows the mean S-SQ per day depending on the study week for both sequence groups. Linear trend lines show the course of S-SQ per week. In both groups, mean S-SQ decreases during the first week. In the second week, i.e., during the CP, sleep quality increases in the I-C group and even slightly exceeds the initial level of the first week. In the C-I group, sleep quality also increases during the second week, but to a lower degree.

### Cortisol and melatonin by condition

Cortisol levels at 14:00 h were higher than levels at 20:00 h in both conditions. The difference between 14:00 and 20:00 h cortisol levels was greater in the IP. Neither difference was statistically significant (Table 2).

**Table 2:** Cortisol and melatonin by condition

	Intervention	Control	Mean difference (95% CI)	*p* value	Effect size
Cortisol 14:00 h (µg/L) (*n* = 9)^a^	1.5 ± 0.7	1.1 ± 0.8	0.4 (− 0.1, 0.9)	0.13	0.51
Cortisol 20:00 h (µg/L) (*n* = 8)^b^	1.0 ± 0.6	1.1 ± 0.8	− 0.2 (− 1.2, 0.9)	0.78	0.10
Melatonin 14:00 h (ng/L) (*n* = 9)^b^	0.3 ± 0.4	0.2 ± 0.3	0.1 (− 0.2, 0.3)	0.72	0.12
Melatonin 20:00 h (ng/L) (*n* = 8)^b^	1.3 ± 1.5	0.3 ± 0.2	1.0 (− 0.3, 2.4)	0.21	0.45
Melatonin mean (14:00 h and 20:00 h) (ng/L) (*n* = 7)^b^	0.9 ± 0.8	0.3 ± 0.1	0.7 (− 0.0, 1.3)	0.06	0.70
Melatonin difference (20:00–14:00 h) (ng/L) (*n* = 7)^b^	0.8 ± 1.5	0.0 ± 0.5	0.9 (− 1.4, 3.1)	0.24	0.45

Values are presented as mean ± SD

^a^Paired *t* test, Hedges correction as effect size

^b^Wilcoxon's test, Pearson’s r as effect size

**Fig. 3:**
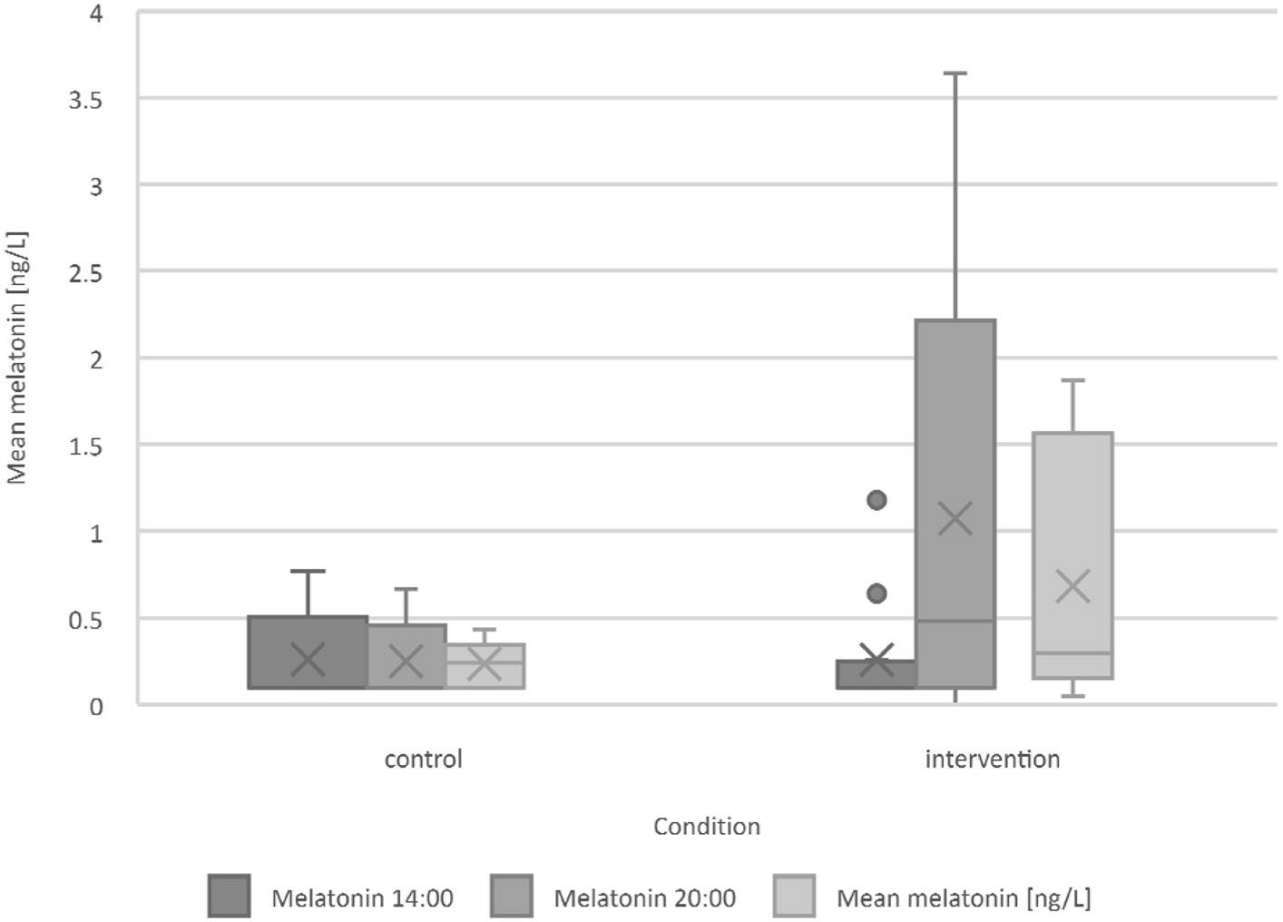
Comparison of mean melatonin levels at 14:00 h, 20:00 h, and on average depending on condition. Melatonin levels are increased during intervention period. Whiskers: 95% CI, x: Mean, o: Outlier

Melatonin levels were higher at both 14:00 h and 20:00 h and on average during the IP (Fig. 3). The difference between midday and evening melatonin levels was also greater during the IP. Neither difference was statistically significant (Table 2).

### O-SQ by condition

SE, SOL, and SFI did not increase significantly in the IP. TST, TSTN, and WASO decreased but not statistically significantly in the IP (SI, “Supplementary file 3”, Table SI-2).

### S-SQ by condition

No statistically significant differences were observed in the mean S-SQ, restfulness, and mood scores and subjective diurnal sleep duration between the CP and IP. (SI, “Supplementary file 3”, Table SI-3).

### Correlation of S-SQ and O-SQ

There was a significant, strong negative correlation between S-SQ and SOL (Spearman’s *ρ* = − 0.502, *p* < 0.047), as well as between S-SQ and WASO (Spearman’s *ρ* = − 0.597, *p* < 0.015). There was no significant correlation between S-SQ and any other O-SQ parameter (SI, “Supplementary file 3”, Table SI-4).

## Discussion

The aim of the present study was to determine whether a daylight intervention in the morning could improve the circadian rhythms of cortisol and melatonin and enhance S-SQ and O-SQ in geriatric patients.

### Acclimatization to the hospital environment

No significant differences were observed in S-SQ and O-SQ between the first and second week of participants' hospital stay (SI, “Supplementary file 3”, Table SI-1). These findings do not support the hypothesis that patients' sleep quality would improve in the course of their hospital stay due to health status improvements [31]. This could be explained by the fact that not all patients exhibited a linear clinical improvement. However, a tendency for S-SQ to deteriorate during the first week and improve again during the second week, irrespective of the condition, could be found (Fig. 2). When the CP was in the second week, S-SQ even slightly exceeded the initial level (6.8) at the end of the second period (7.1). These findings suggest a potential period of acclimatization to the unfamiliar environment and subsequent improvement in sleep [4]. The start of the light intervention in the second week requires readjustment to an unfamiliar environment and may have slowed down the overall progress. This may account for the decrease in S-SQ within the C-I group, from 6.5 at the study's beginning to 5.4 at its end.

### Cortisol

As hypothesized and in agreement with previous studies, midday cortisol levels were found to be higher in the IP compared to the CP, while evening cortisol levels remained the same [32]. However, these results were not statistically significant. The observed cortisol levels were below the expected values of 2.5 µg/L and 1.9 µg/L for healthy older adults at both 14:00 h with 1.5 ± 0.7 µg/L [IP, 1.1 ± 0.8 µg/L (CP)] and at 20:00 h with 1.0 ± 0.6 µg/L [IP, 1.1 ± 0.8 µg/L (CP)] [26]. Due to the seasonal variation of salivary cortisol, we would have expected high cortisol levels in our study, which was conducted exclusively during winter [33]. It is likely that discrepancies between our findings and comparative values can be explained by the used laboratory techniques. While we measured cortisol using liquid chromatography–tandem mass spectrometry, immunoassays were used in comparative studies. In immunoassays, cross-reactivity can lead to falsely high values [34]. This is not the case with the method used here.

### Melatonin

Supporting our hypothesis, mean melatonin levels were higher in the IP than in the CP. The difference in melatonin levels between 14:00 and 20:00 h increased in the IP compared to the CP. Although the observed differences are not statistically significant, it is noteworthy that the interperiodic difference in the melatonin levels at 20:00 h exceeds 1 ng/L [1.33 ng/L (IP), 0.31 ng/L (CP)]. In particular, there is a high variance in the IP. With age, glare sensitivity increases making bright light unpleasant [35]. The actual illumination received by individual participants may have varied due to behavioral reactions related to glare sensitivity, such as repositioning of the lamp and turning away from the light source. Additionally, patients may have inherent issues with light perception, such as those stemming from eye conditions like macular degeneration or cataracts [36, 37]. While all patient files were reviewed for such impairments, it is conceivable that some participants may have had an undiagnosed eye condition.

Similarly to previous studies in geriatric patients [14, 38], this study failed to show significant improvements in circadian rhythms of melatonin, while previous studies have shown beneficial effects of light intervention on melatonin rhythmicity in younger, healthy people [39–41]. This supports that the sensitivity of melatonin to light as a Zeitgeber decreases with age, among other things due to an increasing opacity of the lens [42]. Such a possible insensitivity to light is also reflected in the fact that the melatonin levels at noon are even higher during the IP than during the CP (0.3 ± 0.4 ng/L vs. 0.2 ± 0.3 ng/L), whereas insufficient daylight is thought to increase diurnal melatonin levels [43]. It was observed that melatonin levels remain below expectations at all times. It has been shown previously that there is a seasonal variation in melatonin concentration with particularly low levels in winter [44]. However, the levels observed with mean melatonin levels at 20:00 h of 1.3 ng/L (IP) and 0.3 ng/L (CP) are significantly lower than the levels of 2.1 ng/L in the mean of November and February at 20:00 h in older people expected from previous studies [44]. This could be because our subjects were complex geriatric patients rather than older participants with no health difficulties. In non-healthy older people, a decrease in the mean melatonin concentration has been shown with an increase in diurnal melatonin levels [43, 45]. Even with this approach, our results are significantly lower than in the previous studies in similar subjects. As with the low cortisol levels, this could be explained by the fact that immunoassays were used in comparative studies, where cross-reactivity can lead to falsely high levels, whereas we used liquid chromatography–tandem mass spectrometry.

It is noteworthy that three participants in the C-I group were prescribed new sleep medication during the study period. Melatonin was given to all three patients at 21:00 h, it was administered to two of the three patients only in the middle or towards the end of the second week. They were excluded from any analyses. None from the I-C group were administered new sleep medication. This variation among the sequence groups could suggest that the light intervention was effective in terms of stabilizing the rhythm, as none of the participants who had the IP first required additional sleep medication.

### S-SQ

Previous studies on the effects of a light intervention on S-SQ are inconclusive, with none showing a significant improvement [9]. In contrast to our hypothesis that the daylight intervention would improve S-SQ, a non-significant deterioration from 6.3 at day 1 to 6.0 at day 6 was observed during the IP. As described, behavioral reactions (turning away from light source due to glare perception) and physiological conditions (opacity of lens and thus reduced actual signal) in combination can lead to likely smaller effect of the intervention. Previous studies have shown that the subjective assessment of sleep is heavily influenced by surrounding conditions and how individuals cope with their current situation [17]. Good sleep quality can be observed when individuals adjust to their illness and hospitalization, while bad sleep quality is associated with a sense of helplessness [17]. Nevertheless, S-SQ is also an important parameter to record, as it allows conclusions to be drawn about the overall condition of the patient and is even an independent predictor of mortality in older adults [46].

### O-SQ

The actigraphy results were inconclusive and lacked statistical significance. In contrast to previous studies and our hypothesis, no increase in TST and TSTN could be observed during the IP [14, 38, 47]. Unexpectedly, a high SE could be observed (98.0 ± 2.1% (IP), 97.5 ± 1.6% (CP)) [17, 48]. While wrist-worn actigraphy estimates objective sleep parameters almost correctly compared to polysomnography, hip-worn actigraphy overestimates SE by more than 13% and TST by approximately 14% [49]. If we assume that our results are biased by the same factor due to the measurement method, we can report a value of 86.3% and 85.8% for SE in the IP and CP, respectively and a TST of 322.4 min [IP, 370.1 min (CP)]. Considering the measurement method, our results are consistent with the previous studies in similar populations [17, 48].

### Correlation of S-SQ and O-SQ

In contrast to previous studies, our findings revealed a significant and robust negative correlation between S-SQ and SOL or WASO [17, 18]. This suggests that S-SQ is less influenced by sleep duration than by unintentional wakefulness (insomnia).

## Limitations and strengths

It should be noted that this study also has some limitations.

First, the sample size of 15 participants limits generalizability. Although the sample collected was considered sufficient to demonstrate potential light intervention effects, limitations such as missing saliva samples due to COVID-19 and with data collection methods, did not allow the power needed for the analysis.

Second, exposure to the daylight lamp for 5 h for 6 days during the IP could not be ensured. With increasing sensitivity to glare with age, participants might have felt negatively affected by the lamp possibly leading to behavioral countermeasures. These behaviors were mitigated through regular rounds and adjustments to the lamp with the subjects. In future research, it may be preferable to use non-adjustable light sources and allow subjects to adjust the illuminance themselves, thereby increasing actual exposure by reducing behavioral countermeasures.

Additionally, data from the light sensors could not be used for the main analysis due to methodological limitations that reduced the quality of the data. Further explanation is provided in the supplementary material “Supplementary file 2”.

To enhance the effect of a light intervention on sleep and endocrinological parameters, future studies may consider assessing the circadian rhythm of the subjects prior to the commencement of the study. This addition would allow the timing of the light intervention to be individually adapted to the patient's needs. However, in the case of acutely ill subjects recruited in hospital, it is not feasible to determine hormone levels or O-SQ prior to the study. One potential approach could be to administer questionnaires on chronotype [7]. It should be noted that these do not correlate well with the actual circadian rhythm in patient groups, particularly in older individuals [7, 50].

Conducting studies with acutely ill geriatric patients poses specific challenges that require addressing. Deteriorating health conditions may increase the likelihood of high dropout rates. Furthermore, crossover studies are known to be particularly challenging for patients, as the observation period per subject is twice as long as in a parallel group design [30]. Consequently, study personnel have been intensively deployed to mitigate this challenge. This could not completely overcome the additional burden of study participation in the already stressful situation of being ill and hospitalized.

In future research, it would be beneficial to monitor the subjects’ stress levels and general mood at various points throughout the study, including before, during, and after the study. This procedure would enable the quantification of the stress caused by study participation, hospitalization and the underlying disease. It would also allow for the determination of whether the effectiveness of the light intervention is reduced in people suffering from stress or depressed mood, as sleep quality is linked to mood and vice versa [1, 4]. Furthermore, this addition would allow to analyse whether the light intervention has a positive impact on the mood of the subjects.

On the other hand, the crossover design is also a particular strength of the study. The patients served as their own controls. The dependence of the treatment effects on underlying diseases, pain, and sleep problems can be neglected in this study design, in contrast to a parallel group design. In addition, changes in circadian rhythms, showing large inter-individual differences in older and hospitalized people, can be better compared. It is also possible to assess S-SQ and to compare it between study periods. Adaptive randomization minimized possible effects of the order of the interventions. The 1-day washout period minimized the probability of carry-over effects.

## Conclusions

This study provides preliminary evidence to support that light interventions in the morning might improve geriatric patients’ circadian rhythms of cortisol and melatonin as well as SE and WASO. S-SQ could not be improved by the intervention. In contrast to other studies’ findings, a correlation between SOL or WASO and S-SQ was identified, while sleep duration was found to have no influence on S-SQ. This indicates that some sleep disorders like insomnia might be identified by assessing S-SQ in older adults. Furthermore, one has to pay attention when patients report bad sleep quality as it indicates poor quality of life and is a predictor for mortality [17, 46].

## Supplementary Information

Below is the link to the electronic supplementary material.

Supplementary file 1 (PDF 108 KB)

Supplementary file 2 (PDF 186 KB)

Supplementary file 3 (PDF 208 KB)

## Data Availability

Data are available upon reasonable request.
