# Supplementary material for: Effects of a daylight intervention in the morning on circadian rhythms and sleep in geriatric patients: a randomized crossover trial
Source: Eur Geriatr Med. 2024 Dec 3;16(1):281–92. doi: 10.1007/s41999-024-01100-z (PMC11850413; doi:10.1007/s41999-024-01100-z)
Supplement: Supplementary file 1 — (PDF 108 KB) [file 41999_2024_1100_MOESM1_ESM.pdf]

Effects of a daylight intervention in the morning on circadian rhythms and sleep in geriatric patients: a randomized crossover trial

In: European Geriatric Medicine

Anna Schubert[1], Thea Laurentius, Svenja Lange, Jens Bertram, Leo Cornelius Bollheimer, Marcel Schweiker, Rania Christoforou

[1] Healthy Living Spaces Lab, Institute for Occupational, Social, and Environmental Medicine, Medical Faculty, RWTH Aachen University, 52074 Aachen, Germany. Corresponding author: anna.schubert@rwth-aachen.de

Sleep questionnaires on day 5 and 6

On day 5 of each period respectively – when the 20:00 h saliva sample was taken – participants were asked to complete the following questionnaire (in its German version) at 20:00 h:

1. Did you sleep today? If so, how long? When?
2. Were you outside at daylight today? If so, how long? When?
3. Did you take any sleeping aid today?

On day 6 of each period respectively they were asked to complete the following questionnaire in the morning at 8:00 h:

1. How restful was your sleep? (Rated on a scale of 1 (meaning not restful at all) to 5 (meaning very restful))
2. How are you feeling now? (Rated on a scale of 1 (meaning depressed) to 5 (meaning lighthearted))

The questions were created based on the sleep diary of Charité - Universitätsmedizin Berlin (https://schlaf.charite.de/fileadmin/user_upload/microsites/kompetenzzentren/schlaf/Schlaftagebuch.pdf).
