# Supplementary material for: Effects of a daylight intervention in the morning on circadian rhythms and sleep in geriatric patients: a randomized crossover trial
Source: Eur Geriatr Med. 2024 Dec 3;16(1):281–92. doi: 10.1007/s41999-024-01100-z (PMC11850413; doi:10.1007/s41999-024-01100-z)
Supplement: Supplementary file 2 — (PDF 186 KB) [file 41999_2024_1100_MOESM2_ESM.pdf]

Effects of a daylight intervention in the morning on circadian rhythms and sleep in geriatric patients: a randomized crossover trial

In: European Geriatric Medicine

Anna Schubert[1], Thea Laurentius, Svenja Lange, Jens Bertram, Leo Cornelius Bollheimer, Marcel Schweiker, Rania Christoforou

[1] Healthy Living Spaces Lab, Institute for Occupational, Social, and Environmental Medicine, Medical Faculty, RWTH Aachen University, 52074 Aachen, Germany. Corresponding author: anna.schubert@rwth-aachen.de

Limitations of illuminance data recording

Illuminance meters often measured an illuminance of 0lx even during daytime. During the study period, patients were observed to put on further pieces of cloth, potentially to overcome a feeling of cold as possible during wintertime. Despite conducting routine checks and making necessary adjustments to the sensors and patient training, such clothing adjustments must have covered the sensors and could not be prevented sufficiently. To illustrate the problem with data recording by the light sensors, the collected data were truncated to the period from 8:00 h to 13:00 h on study days 2 to 7 and 9 to 14 and then divided into two data quality groups "good" and "poor". One would expect a high median illuminance during both periods and a higher median and mean illuminance during the intervention period. Subjects showing this pattern were assigned to the "good" data quality group (n=5). Extended periods of no exposure (mean illuminance of 0lx) and higher mean illuminance during the control period indicate poorer data quality. Subjects showing this pattern were assigned to the “poor” data quality group (n=7). Subjects who did not meet either definition or had deviations from the study plan that would have biased the results were excluded from the analysis (n=6). A comparison of illuminance by data quality and period is shown in Figures 1 and 2.

Fig. 1
Illuminance by data quality and period (scale up to 30000lx) from 8:00 h to 13:00 h

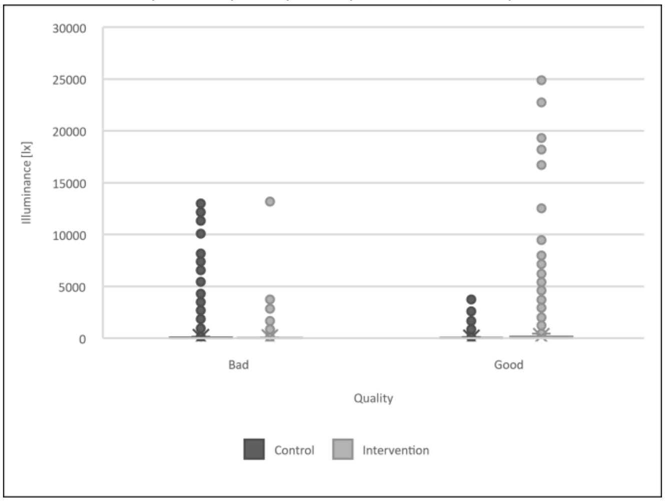

Whiskers: 95% CI, x: Mean

Fig. 2

Illuminance by data quality and period (scale up to 500lx) from 8:00 h to 13:00 h

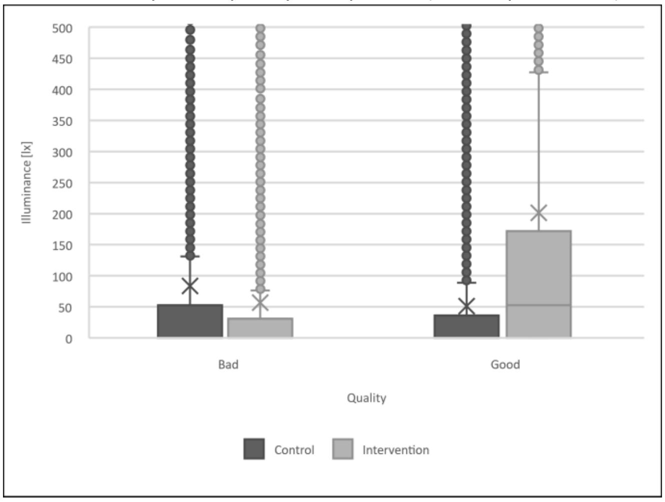

Whiskers: 95% CI, x: Mean
