# Supplementary material for: Effects of a daylight intervention in the morning on circadian rhythms and sleep in geriatric patients: a randomized crossover trial
Source: Eur Geriatr Med. 2024 Dec 3;16(1):281–92. doi: 10.1007/s41999-024-01100-z (PMC11850413; doi:10.1007/s41999-024-01100-z)
Supplement: Supplementary file 3 — (PDF 208 KB) [file 41999_2024_1100_MOESM3_ESM.pdf]

Effects of a daylight intervention in the morning on circadian rhythms and sleep in geriatric patients: a randomized crossover trial

In: European Geriatric Medicine

Anna Schubert[1], Thea Laurentius, Svenja Lange, Jens Bertram, Leo Cornelius Bollheimer, Marcel Schweiker, Rania Christoforou

[1] Healthy Living Spaces Lab, Institute for Occupational, Social, and Environmental Medicine, Medical Faculty, RWTH Aachen University, 52074 Aachen, Germany. Corresponding author: anna.schubert@rwth-aachen.de

Tables

Table SI-1
Results for period effects

	Control-Intervention		Intervention-Control		Mean difference (95% CI)	*p*-value
Cortisol 14:00 h [µg/L][a]	n=4	0.3 ± 0.6	n=5	-0.5 ± 0.8	0.7 (-0.4, 1.9)	.164
Cortisol 20:00 h [µg/L][a]	n=2	0.0 ± 0.7	n=6	0.2 ± 1.4	-0.3 (-2.9, 2.4)	.823
Melatonin 14:00 h [ng/L][a]	n=4	0.0 ± 0.5	n=5	-0.1 ± 0.2	0.1 (-0.3, 1.8)	.749
Melatonin 20:00 h [ng/L][a]	n=2	1.8 ± 2.2	n=6	-0.8 ± 1.6	2.5 (-0.9, 6.0)	.122
Melatonin mean (14:00 h and 20:00 h) [ng/L][a]	n=2	0.9 ± 0.7	n=5	-0.6 ± 0.8	1.5 (-0.3, 3.2)	.084
Melatonin difference (20:00 h-14:00 h) [ng/L][a]	n=2	1.8 ± 2.9	n=5	-0.9 ± 1.6	2.7 (-1.5, 6.9)	.156
SE [%][a]	n=4	0.3 ± 0.9	n=4	-0.7 ± 1.0	1.0 (-0.7, 2.6)	.198
SOL [minutes][b]	n=4	0.1 ± 0.2	n=4	0.0 ± 0.1	0.1 (-0.2, 0.4)	.686
TST [minutes][a]	n=4	-7.8 ± 34.5	n=4	65.2 ± 170.0	-73.0 (-285.2, 139.2)	.432
TSTN [minutes][a]	n=4	-18.2 ± 71.0	n=4	71.4 ± 163.7	-89.6 (-308.0, 128.7)	.354
TSTD [minutes][a]	n=4	16.4 ± 85.3	n=4	-121.0 ± 158.1	137.4 (-82.3, 357.2)	.177
WASO [minutes][a]	n=4	-0.4 ± 3.0	n=4	4.1 ± 3.0	-4.5 (-9.7, 0.6)	.075
SFI [-][a]	n=4	-2.0 ± 7.6	n=4	-4.9 ± 11.2	2.8 (-13.7, 19.4)	.689
Subjective sleep quality mean [-] (day 1 to day 6)[b]	n=6	0.2 ± 1.2	n=9	0.9 ± 1.1	-0.6 (-1.9, 0.7)	.272
Restfulness [-][a]	n=3	0.0 ± 1.0	n=6	0.2 ± 0.8	-0.2 (-1.6, 1.2)	.785
Feeling [-][a]	n=3	0.0 ± 1.0	n=6	0.3 ± 1.6	-0.3 (-2.8, 2.1)	.759
Subjective diurnal sleep duration [minutes][b]	n=6	-3.0 ± 65.9	n=9	-14.5 ± 47.2	11.5 (-51.3, 74.3)	.864

Values are presented as mean ± SD. [a]Independent two sample t-test, [b]Mann-Whitney U test (exact significance reported)
SE = sleep efficiency, SOL = sleep onset latency, TST = total sleep time, TSTN = nocturnal total sleep time, TSTD = diurnal total sleep time, WASO = wakefulness after sleep onset, SFI = sleep fragmentation index

Table SI-2

Objective sleep quality by condition

	Intervention	Control	Mean difference (95% CI)	*p*-value	Effect size
SE [%] (n=8)[a]	98.0 ± 2.1	97.5 ± 1.6	0.5 (-0.3, 1.2)	.123	.544
SOL [minutes] (n=8)[a]	0.1 ± 0.2	0.0 ± 0.1	0.1 (-0.1, 0.2)	.285	.378
TST [minutes] (n=8)[b]	368.2 ± 74.7	422.7 ± 91.3	-36.5 (-134.8, 61.8)	.409	.276
TSTN [minutes] (n=8)[b]	405.0 ± 102.3	449.9 ± 91.0	-44.8 (-145.3, 55.7)	.327	.331
TSTD [minutes] (n=8)[a]	307.9 ± 202.2	239.1 ± 204.8	68.7 (-40.2, 177.6)	.345	.333
WASO [minutes] (n=8)[a]	7.9 ± 7.9	10.2 ± 6.2	-2.3 (-5.1, 0.6)	.123	.544
SFI [-] (n=8)[b]	25.5 ± 5.1	24.1 ± 9.1	1.4 (-6.6, 9.5)	.688	.131

Values are presented as mean ± SD. [a]Wilcoxon's test, Pearson’s r as efffect size. [b]Paired t-test, Hedges correction as effect size.
SE = sleep efficiency, SOL = sleep onset latency, TST = total sleep time, TSTN = nocturnal total sleep time, TSTD = diurnal total sleep time, WASO = wakefulness after sleep onset, SFI = sleep fragmentation index

Table SI-3

Subjective parameters by condition

	Intervention	Control	Mean difference (95% CI)	*p*-value	Effect size
Subjective sleep quality mean [-] (day 1 to day 6) (n=15)[a]	6.1 ± 1.6	6.5 ± 1.5	-0.4 (-1.1, 0.3)	.207	.323
Restfulness [-] (n=9)[a]	3.6 ± 1.0	3.7 ± 1.4	-0.1 (-0.7, 0.5)	.681	.128
Mood [-] (n=9)[b]	3.8 ± 1.2	4.0 ± 1.3	-0.2 (-1.3, 0.6)	.603	.173
Subjective diurnal sleep duration [minutes] (n=15)[b]	37.8 ± 45.2	30.3 ± 40.0	7.5 (-22.3, 37.4)	.624	.127

Values are presented as mean ± SD. [a]Paired t-test, Hedges correction as effect size. [b]Wilcoxon's test, Pearson’s r as efffect size.

Table SI-4

Correlation of subjective and objective sleep quality

	Subjective sleep quality mean (day 1 to day 6)	
	**Spearman's *ρ***	***p*-value**
SE	.472	.065
SOL	-.502	.047
TST	.010	.970
TSTN	-.024	.931
TSTD	-.74	.786
WASO	-.597	.015
SFI	-.024	.931

SE = sleep efficiency, SOL = sleep onset latency, TST = total sleep time, TSTN = nocturnal total sleep time, TSTD = diurnal total sleep time, WASO = wakefulness after sleep onset, SFI = sleep fragmentation index
